# Insights into the Pathology of the α_2_-Na^+^/K^+^-ATPase in Neurological Disorders; Lessons from Animal Models

**DOI:** 10.3389/fphys.2016.00161

**Published:** 2016-05-04

**Authors:** Toke J. Isaksen, Karin Lykke-Hartmann

**Affiliations:** ^1^Department of Biomedicine, Aarhus UniversityAarhus, Denmark; ^2^Centre for Membrane Pumps in Cells and Disease-PUMPKIN, Danish National Research Foundation, Department of Molecular Biology and Genetics, Aarhus UniversityAarhus, Denmark; ^3^Aarhus Institute of Advanced Studies, Aarhus UniversityAarhus, Denmark

**Keywords:** α_2_ sodium ion pump, astrocytes, familial hemiplegic migraine type 2 (FHM2), mouse models

## Abstract

A functional Na^+^/K^+^-ATPase consists of a catalytic α subunit and a regulatory β subunit. Four α isoforms of the Na^+^/K^+^-ATPase are found in mammals, each with a unique expression pattern and catalytic activity. The α_2_ isoform, encoded by the *ATP1A2* gene, is primarily found in the central nervous system (CNS) and in heart-, skeletal- and smooth muscle tissues. In the CNS, the α_2_ isoform is mainly expressed in glial cells. In particular, the α_2_ isoform is found in astrocytes, important for astrocytic K^+^ clearance and, consequently, the indirect uptake of neurotransmitters. Both processes are essential for proper brain activity, and autosomal dominantly mutations in the *ATP1A2* gene cause the neurological disorder Familial hemiplegic migraine type 2 (FHM2). FHM2 is a severe subtype of migraine with aura including temporary numbness or weakness, and affecting only one side of the body. FHM2 patients often suffer from neurological comorbidities such as seizures, sensory disturbances, cognitive impairment, and psychiatric manifestations. The functional consequences of FHM2 disease mutations leads to a partial or complete loss of function of pump activity; however, a clear phenotype-genotype correlation has yet to be elucidated. Gene-modified mouse models targeting the *Atp1a2* gene have proved instrumental in the understanding of the pathology of FHM2. Several *Atp1a2* knockout (KO) mice targeting different exons have been reported. Homozygous *Atp1a2* KO mice die shortly after birth due to respiratory malfunction resulting from abnormal Cl^−^ homeostasis in brainstem neurons. Heterozygous KO mice are viable, but display altered behavior and neurological deficits such as altered spatial learning, decreased motor activity and enhanced fear/anxiety compared to wild type mice. FHM2 knock-in (KI) mouse models carrying the human *in vivo* disease mutations W887R and G301R have also been reported. Both models display altered cortical spreading depression (CSD) and point to deficits in the glutamatergic system as the main underlying mechanism of FHM2.

## The Na^+^/K^+^-ATPase: expression and function

The Na^+^/K^+^-ATPase is a transmembrane ion-pump located at the plasma membrane of all mammalian cells. It's function moves three Na^+^ ions out of the cell and two K^+^ ions into the cell utilizing energy from ATP hydrolysis (Skou, 1957).

A functional pump consists of a catalytic α-subunit and a regulatory β-subunit (Kaplan, 2002). Four α-subunit isoforms, designated as α_1_-α_4_, encoded by different genes are found in mammals. Each α-subunit isoform has its own unique expression pattern and catalytic activity, which can be modulated by the β-subunit (Larsen et al., 2014; Hilbers et al., 2016). The *ATP1A1* gene encodes the α_1_isoform, which is considered to be the isoform that maintains basic cellular functions and is accordingly expressed almost ubiquitously in all tissue and cell types (Lingrel et al., 2007). The α_2_ isoform, encoded by the *ATP1A2* gene, is primarily expressed in the central nervous system (CNS) and in heart-, skeletal,- and smooth muscle tissues. In the CNS, the α_2_ isoform is expressed primarily in astrocytes but also in other glial cells (McGrail et al., 1991). During embryonic development, the α_2_ isoform is expressed primarily in neurons, which gradually changes to the glial cell expression pattern in the adult, murine brain (McGrail et al., 1991; Cameron et al., 1994; Cholet et al., 2002; Moseley et al., 2003).

The α_3_ isoform, encoded by the *ATP1A3* gene, is also expressed in the CNS and nervous tissue, but expresses specifically in the neurons of the basal ganglia, cerebellum, and hippocampus (McGrail et al., 1991; Bøttger et al., 2011; Li et al., 2013).

General Na^+^/K^+^-ATPase activity is crucial for multiple cellular functions such as maintaining resting membrane potential, regulating cellular volume, regulating pH, and driving the secondary active transport (Dobretsov and Stimers, 2005). Regulated and specialized pump activity is important for normal brain function. This is also reflected in the expressive relationship between the α_1_, α_2_, and α_3_ isoforms in the CNS and the complex neurological disorders caused by mutations in the *ATP1A2* and *ATP1A3* genes (reviewed in Bøttger et al., 2012; Heinzen et al., 2014). Furthermore, increased evidence suggests roles for the Na^+^/K^+^-ATPase in regulating signaling pathways, such as: the membrane-associated non-receptor tyrosine kinase Src: activation of Ras/Raf/ERK1,2, phosphatidylinositol 3-kinase (PI3K): PI3K-dependent protein kinase B, phospholipase C, (${\text{Ca}}_{2}^{+}$)_i_ oscillations (Aperia, 2007; Schoner and Scheiner-Bobis, 2007; Liu and Xie, 2010): and gene transcription (Egr-1, Fos, Jun, Nr4a2, Hes1, and Gabre; Tupler et al., 2001).

## The α_2_ isoform and its function in astrocytes

Astrocytes are some of the most abundant cells in the CNS of mammals, with a ratio of 1.4 astrocytes for every neuron in the human cortex (Nedergaard et al., 2003). Through their physical properties and neuron-glial signaling pathways, they play an essential role in neuronal homeostasis through their physical properties and neuron-glial signaling pathways. During neuronal activity, a vast efflux of K^+^ ions into the extracellular space occurs. The extracellular concentration of K^+^ ranges from 2.5 to 3.5 mM in normal conditions but can increase to 50–80 mM under ischemic and cortical spreading depression events (Walz, 2000).

Since imbalances between extracellular K^+^ and K^+^ clearance can affect neuronal excitability and abnormal brain function as well as cause significant neuronal death. Even under conditions of abundant glucose supply, extracellular K^+^ homeostasis must be tightly regulated to support normal brain activity (Walz, 2000).

Spatial buffering plays a critical role in the mechanism of K^+^ clearance (Karwoski et al., 1989; Walz, 2000; Kofuji and Newman, 2004). This principle is based on the influx of K^+^ into astrocytes though K^+^ channels, with the central channel being K_ir_4.1, a member of the inward rectifier-type potassium channel family. This positive influx then spreads electronically through the cytoplasm of the astrocyte and exits again as K^+^ at locations distant from the active neurons (Karwoski et al., 1989; Kofuji and Newman, 2004; Macaulay and Zeuthen, 2012). In addition to K_ir_4.1, both the Na^+^/K^+^-ATPase and the Na^+^/K^+^/2Cl^−^ cotransporter 1 (NKCC1) are necessary for the import and export of K^+^ in the astrocytes (Galvan et al., 1979; D'Ambrosio et al., 2002; MacVicar et al., 2002; reviewed in Walz, 2000; Hertz et al., 2015). This is further described elsewhere in this special issue by MacAulay and colleagues.

In the Na^+^/K^+^-ATPase, the α_2_ isoform combined with the β_2_ subunit have K^+^ affinity and voltage-sensitivity. As a result it is specifically geared to control extracellular K^+^ concentration during intense neuronal firing (Larsen et al., 2014). Furthermore, the α_2_ and β_2_ subunit isoforms are co-expressed and appears to co-localize in astrocytes in the rodent brain (McGrail et al., 1991; Fink et al., 1996; Knapp et al., 2000; Cholet et al., 2002). Thus, the α_2_/β_2_ complex appears to be the main Na^+^/K^+^-ATPase constellation involved in the astrocytic K^+^ clearance (Larsen et al., 2014).

Controlling the neurotransmitter levels present in the synapse is required for functional neuronal signaling, plasticity and neuroprotection. Removal of neurotransmitters occurs through diffusion, enzymatic degradation, and the reuptake by neurons and glial cells. The importance of astrocytic neurotransmitter uptake is highlighted in mice deficient of the astrocytic glutamate transporter excitatory amino acid transporter 2 (EAAT2), where *Eatt2*-deficiency causes lethal spontaneous seizures and increased susceptibility to acute cortical injury in mice (Tanaka et al., 1997). The α_2_ isoform localizes predominately in astrocytes and other glial cells (McGrail et al., 1991; Fink et al., 1996; Knapp et al., 2000; Cholet et al., 2002) and has been shown to co-distribute with both astrocytic glutamate transporters excitatory amino acid transporter 1 (EAAT1) and EAAT2 (Cholet et al., 2002). Additionally, the α_2_ isoform immunoprecipitated with both EAAT1 and EAAT2 in rat cerebellar and forebrain tissues, respectively (Rose et al., 2009). In the same study, it was shown that the ouabain-mediated inhibition of Na^+^/K^+^-ATPase activity had an inhibitory effect on glutamate uptake. Another study similarly found the pump combination α_2_/β_2_ in a protein complex with glutamate and lactate transports, thereby sustaining glutamate-dependent lactate transport (Kleene et al., 2007). The uptake of glutamate via glutamate transporters is driven by the Na^+^-gradient. Astrocytes, therefore, rely on Na^+^,K^+^-ATPases to pump out accumulated Na^+^ (Grewer and Rauen, 2005). The direct interaction between the α_2_ isoform and astrocytic glutamate transporters highlights this dependency, suggesting the existence of local regulation and crosstalk between Na^+^/K^+^-ATPases activity and glutamate uptake. Based on the dependency of many other neurotransmitter transporters on the Na^+^-gradient, it is likely that the Na^+^/K^+^-ATPase is also functionally linked to these transporters. Numerous studies have shown a coupling between the α_3_ subunit and neurotransmitter transport such as the glycine transporter GlyT2 (de Juan-Sanz et al., 2013).

## Familial hemiplegic migraine

Familial hemiplegic migraine (FHM) is an autosomal dominant inherited migraine with aura and typically includes episodes of temporary numbness or weakness affecting one side of the body (hemiparesis). There are three recognized subtypes of FHM (FHM1-3), each caused by distinctive mutations in a specific gene. FHM Type 1, which accounts for around 50% of all FHM cases, is caused by mutations in the *CACNA1A* gene encoding the Ca_v_2.1 P/Q voltage-dependent calcium channel (Ophoff et al., 1996). FHM Type 2 is caused by mutations in the *ATP1A2* gene (De Fusco et al., 2003) and FHM type 3 is caused by mutations in the *SCN1A* gene encoding the Na^+^ channel, voltage-gated, type I, α subunit (Dichgans et al., 2005). Thus, all three types of FHM are related to alterations in ion-transport and ion-homostasis.

FHM patients in general share common migraine associated symptoms such as nausea, vomiting, and increased sensitivity to stimuli such as sound and light. In addition, diverse comorbidities have in addition been observed for each subtype and also for different mutations in a given subtype. In FHM2, observed neurological symptoms include sensory disturbance, impaired vision during aura episodes, speech difficulties, varied forms of epilepsy and seizures, developmental disabilities, cognitive impairments and cerebellar defects, which cause ataxia, dysarthria, and nystagmus. Rarer manifestations such as psychiatric disorders (depression, borderline personality, and obsessive-compulsive disorder), obesity, dystonic posturing, and anxiety have also been reported (Barrett et al., 2008; Bøttger et al., 2012; Ferrari et al., 2015).

The genetic linkage between *ATP1A2* and FHM2 was first made in 2003 (Marconi et al., 2003). In this study of two large Italian families with extensive history of FHM2, the FHM2 locus was narrowed to a 0.9 Mb region on chromosome 1q23 wherein mutations in the *ATP1A2* gene were first identified. Subsequent sequence analysis of candidate genes revealed specific FHM2-associated mutations in the *ATP1A2* gene in each of the families (De Fusco et al., 2003). Numerous new mutations in the *ATP1A2* gene have been reported post-analysis, indicating that there are more than 35 FHM2-associated mutations, spanning most of the *ATP1A2* coding region (Bøttger et al., 2012). Approximately half of the FHM2 mutations tested *in vitro* show complete loss of pump function, while a majority of the remainder showed reduced pump activity relative to normal α_2_ pump activity. A small number of mutations either prevent α_2_ expression or prevent the transport of α_2_ to the plasma membrane (Bøttger et al., 2012).

To date, genotype-phenotype correlations have yet to be made, yet it is likely that the severity of the main symptoms and the spectra of comorbidities are linked to the effect of the mutation.

Due to the heterogeneity of human patients, however, it may be impossible to correlate genotype-phenotype by clinical patients studies. Therefore animal models are important tools for understanding the spectrum of FHM2 phenotypes and to elucidate the mechanisms behind FHM2.

## Gene-modified α_2_ mouse models

Several gene-modified mouse models targeting the *Atp1a2* gene have been reported, and extensively used to study the *in vivo* functions of the α_2_ isoform (Table 1).

**Table 1:** ***Atp1a2* gene modified mouse models**.

Mouse model^*^	***Atp1a2* genetic alteration**	**Major behavioral observations**	**References**
α_2_^+/KOE4^	Deletion targeting exon 4	- Enhanced fear and anxiety - Hypoactivity - Impaired spatial learning	James et al., 1999; Lingrel et al., 2007; Moseley et al., 2007
α_2_^+/KOE21^	Deletion targeting exon 21	- Enhanced fear and anxiety	Ikeda et al., 2003
α_2_^+/KOE2^	Deletion targeting exon 2	- Not assessed	Ikeda et al., 2004
α_2_^+/W887R^	Single nucleotide substitution (T2763C) causing a single amino acid substitution (W887R)	- Enhanced fear and anxiety	Leo et al., 2011
α_2_^+/G301R^	Single nucleotide substitution (G901A) causing a single amino acid substitution (G301R).	- Hypoactivity (females only) - Compulsive behaviors (females only) - Stress-induced depression	Bøttger et al., 2016

* *Three Atp1a2 knock-out (KO) and two knock-in (KI) mice have been described. For all five models, only heterozygous animals are viable after birth. No models of conditional Atp1a2 KO in CNS related cells have been reported*.

Only heterozygous α_2_ knock-out (KO) and knock-in (KI) mice are viable, as homozygous mice dies immediately after birth (James et al., 1999; Ikeda et al., 2003, 2004; Leo et al., 2011). These immature deaths of homozygous mice were reportedly caused by respiratory failure in α_2_^KOE2/KOE2^ mice (Ikeda et al., 2004). The activity of respiratory motor neurons in the fourth cervical ventral root were altered in α_2_^KOE2/KOE2^ fetuses, as the response of these neurons to electrical stimulation in the ventrolateral medulla was significantly slower in homozygous KO fetuses compared to wild type fetuses (Ikeda et al., 2004). A higher intracellular Cl^−^ concentration was found in these ventrolateral medulla neurons in α_2_^KOE2/KOE2^ fetuses. Furthermore, the α_2_ isoform co-immunoprecipitated with the K^+^-Cl^−^ cotransporter KCC2, suggesting a functional coupling (Ikeda et al., 2004; Pedersen et al., 2006). This is supported by the fact that homozygous *Kcc2* KO mice also die immediately after birth due to severe motor deficits that abolish respiration (Hübner et al., 2001). Thus, homozygous α_2_^KOE2/KOE2^ mice with a complete loss of the α_2_ isoform function, exhibit severe deficits in Cl^−^ homeostasis in vulnerable cells including the respiratory center neurons, thereby causing abnormal neuronal activity and resulting in respiratory failure upon birth.

## α_2_ isoform knock-out mice

Relative to WT littermates, heterozygous α_2_^+/KOE21^ and α_2_^+/KOE4^ mice showed a 50% reduction in the α_2_ isoform protein levels (Ikeda et al., 2003; Moseley et al., 2007). The α_1_ isoform protein levels were comparable between the two groups of mice, whereas α_3_ isoform protein levels in α_2_^+/KOE4^ mice were reduced to 80% relative to WT (Moseley et al., 2007).

Both α_2_^+/KOE4^ and α_2_^+/KOE21^ mice models displayed increased fear and anxiety behavior as the main abnormal behavioral phenotype (Ikeda et al., 2003; Lingrel et al., 2007; Moseley et al., 2007). Characteristic of these behavioral phenotypes, the α_2_^+/KOE21^ mice had fewer entries and spent less time in the open arms in the elevated plus maze test (Ikeda et al., 2003). In a similar zero maze test screening for fear and anxiety, the same pattern was observed in in α_2_^+/KOE4^ mice (Moseley et al., 2007). Another common anxiety behavioral test is the light/dark test, which consists of one illuminated room connected to one dark room. The mouse to be tested is placed in the dark room. The time of first entry into the illuminated room and all subsequent periods of time spent in this room are recorded. Compared to WT littermates, α_2_^+/KOE21^ mice not only spent significantly less time in the illuminated room, but the latency to enter the illuminated room was also significantly higher (Ikeda et al., 2003), indicating increased fear and anxiety behavior phenotypes.

In the open field test, α_2_^+/KOE4^ mice were found to be overall less active compared to WT mice (Moseley et al., 2007). In contrast, no altered spontaneous activity in home cage was observed for the α_2_^+/KOE21^ mice (Ikeda et al., 2003). This suggests that the hypolocomotion observed in the open field test was induced by the enhanced stress and anxiety response in α_2_^+/KOE4^ mice, caused by the unfamiliar and open environment of the open field setup. This correlates with the observation that α_2_^+/KOE4^ mice spend more time in the periphery region instead of the center region during the open field test, which is a commonly used indicator of enhanced fear behavior (Moseley et al., 2007).

Utilizing the Morris water maze test, α_2_^+/KOE4^ mice were also tested for spatial learning and memory (Moseley et al., 2007). The latency to find the hidden platform was higher for α_2_^+/KOE4^ mice compared to WT, which normally indicates impaired learning. However, the distance traveled to reach the platform was comparable for α_2_^+/KOE4^ and WT mice, suggesting no impairment of spatial memory (Moseley et al., 2007). Thus, the increased latency could be a consequence of the enhanced fear and anxiety related behavior observed in α_2_^+/KOE4^ mice, as the immersion in water is highly stressful to the mouse. In support of this, the α_2_^+/KOE21^ mice display an increased freezing time in conditioned fear stimuli, which suggests that learning and memory capability is not impaired (Ikeda et al., 2003).

The enhanced fear and anxiety behavior was investigated further in α_2_^+/KOE21^ mice and homozygous α_2_^KOE21/KOE21^ fetuses. It was found that homozygous α_2_^KOE21/KOE21^ fetuses displayed selective neuronal apoptosis in the amygdala and piriform cortex, both regions involved in fear, anxiety and basic defense mechanism behavior (Davis et al., 1994; Ikeda et al., 2003). Furthermore, c-Fos expression was significantly elevated in these regions in α_2_^KOE21/KOE21^ fetuses and also in adult α_2_^+/KOE21^ mice after conditioned fear stimuli, which indicate neuronal hyperactivity in the areas (Ikeda et al., 2003). An increase in the excitability of amygdala output neurons increased aversive fear conditioning (Davis et al., 1994), thus the enhanced fear and anxiety behavior of α_2_ KO mice probably arise from abnormal function of these brain regions.

Uptake of glutamate and GABA into crude synaptosome preparations from α_2_^KOE21/KOE21^ fetuses was impaired compared to WT preparations and consequently, both glutamate and GABA levels were increased in brains from α_2_^KOE21/KOE21^ fetuses relative to WT littermates (Ikeda et al., 2003). A dysfunction in the removal of neurotransmitters from synapse could be the underlying cause of neuronal hyperactivity and of the observed neurodegeneration in the amygdala and piriform cortex. This is further supported by other studies showing co-localization, molecular interaction and functional interaction between α_2_ and glutamate transporters (Cholet et al., 2002; Kleene et al., 2007; Rose et al., 2009). It is reasonable to suggest that heterozygous mice also share these abnormalities, which cause the observed behavioral phenotypes in α_2_^+/KOE21^ mice. Another plausible explanation, as observed in α_2_^KOE2/KOE2^ fetuses, is the α_2_ isoform interaction with KCC2 and altered intracellular Cl^−^ concentration, which appears to be the cause of respiratory failure in α_2_^-/-^ mice (Ikeda et al., 2004; Pedersen et al., 2006). This Cl^−^ homeostasis deficit could result in enhanced fear and anxiety behavior for all α_2_^+/-^ mice. Similar to the α_2_^+/KOE4^ and α_2_^+/KOE21^ mice, hypomorphic mice with 17% KCC2 levels display increased anxiety-like behavior in several behavioral tests, including the elevated-plus maze test (Ikeda et al., 2003; Tornberg et al., 2005; Moseley et al., 2007).

While most neurological studies involving the α_2_ subunit focus on its effect in FHM2, several recent studies have also linked the Na^+^/K^+^-ATPase to other critical neurological disorders such as Amyotrophic lateral sclerosis (ALS), Huntington's disease and Alzheimer's disease (Acuña et al., 2013; Valencia et al., 2013; Gallardo et al., 2014; Ohnishi et al., 2015). In the ALS study, the α_2_^+/KOE4^ model was used to study the pathology of this disease. A protein complex consisting of α_2_ and α-adducin was found enriched in cultured astrocytes expressing mutant superoxide dismutase 1 (SOD1), the most common genetic factor for ALS (Gallardo et al., 2014). Interestingly, knock down of α_2_ in cultured astrocytes protected co-cultured motor neurons from degeneration, and mating α_2_^+/KOE4^ and SOD1^G93A^ mutant mice significantly reduced neurodegeneration, thereby increasing the lifespan of α_2_^+/KOE4-^ SOD1^G93A^ mice compared to SOD1^G93A^ mice (Gallardo et al., 2014).

## α_2_ isoform knock-in mice

Western blotting for α_2_ isoform protein in brain lysates showed reduced α_2_ protein levels to around 60% in heterozygous adult α_2_^+/W887R^ (Leo et al., 2011) and α_2_^+/G301R^ mice (Bøttger et al., 2016) and to very minimal levels in homozygous fetuses of α_2_^G301R/G301R^. Specific brain regions were additionally probed in the α_2_^+/G301R^ mice with similar results (Bøttger et al., 2016). No alteration in the α_1_ and α_3_ subunit isoform protein levels was found in either of the KI mouse models (Leo et al., 2011; Bøttger et al., 2016), suggesting that these isoforms are not upregulated to compensate for the haploinsufficiency of the α_2_ subunit in the α_2_^+/W887R^ and α_2_^+/G301R^ mice.

Mutated α_2_-W887R transfected into HeLa cells was expressed but did not properly transport to the cell surface. Instead, it was found confined to the endoplasmic reticulum and proteasome inhibition with MG132 increased α_2_-W887R levels significantly (Leo et al., 2011). This suggests improper folding and subsequently proteasomal degradation as the fate of α_2_-W887R. However, transfection of α_2_-W887R into COS7 cells, showed α_2_-W887R at the plasma membrane, by both immunofluorescence staining and by subcellular fractionation (De Fusco et al., 2003). Interestingly, α_2_-W887R located to the plasma membrane when expressed in *Xenopus laevis* oocytes (Koenderink et al., 2005). On measuring ion flux and ATPase activity, this W887R mutation was shown to be inactive (Koenderink et al., 2005).

*In vitro* studies on α_2_-G301R found that this mutation did not cause significant changes in mRNA stability as mRNA levels in HeLa cells transfected with either α_2_ or α_2_-G301R, respectively, were comparable (Santoro et al., 2011). However, immunofluorescence staining for transfected α_2_-G301R showed that the mutated version did not express properly. Thus, it was concluded that this loss of function mutation prompts deficits in folding, maturation, or intracellular trafficking of the α_2_-G301R that causes it to be degraded (Santoro et al., 2011). From these studies and the reduced α_2_ levels in α_2_^+/W887R^ and α_2_^+/G301R^ mice, it appears reasonable to consider both mutated pumps expressed but display no functional pump activity due to improper transport and/or degradation. This is an important difference to the KO models, since mutated α_2_ protein (α_2_-W887R and α_2_-G301R) might still be able to interact with important factors such as the β subunits that could have an effect on the unmutated α_2_ isoform in these models.

The phenotypic behavioral consequences of introducing the W887R mutation were assessed using a modified SHIRPA protocol (Leo et al., 2011). In this protocol, few behavioral differences were observed between α_2_^+/W887R^ and WT mice. The only significant alteration between α_2_^+/W887R^ mice compared to WT littermates was an elevated fear response and increased anxiety (Leo et al., 2011).

Behavioral phenotypes were likewise addressed in the α_2_^+/G301R^ mice. During the tail suspension test, for example, the α_2_^+/G301R^ mice exhibited depression-like behavior, such as increased immobility, compared to WT mice (Bøttger et al., 2016). In a sucrose preference test, α_2_^+/G301R^ mice displayed stress-induced anhedonia and increased acoustic startle responses, implying abnormal levels of fear and anxiety (Bøttger et al., 2016). Female α_2_^+/G301R^ mice were found to have additional abnormal behavioral phenotypes compared to male α_2_^+/G301R^ mice. Unlike WT females and male α_2_^+/G301R^ mice, α_2_^+/G301R^ females were hypoactive in open field test (Bøttger et al., 2016). Excessive grooming behaviors were also observed in the open field, suggesting compulsive behavior in female α_2_^+/G301R^ mice. The marble-burying test is one of the standard behavioral tests for rodents to assess repetitive compulsive-like behaviors. Female α_2_^+/G301R^ mice were found to display obsessive compulsive disorder (OCD)-like traits as they buried significantly more marbles compared to both WT and to male α_2_^+/G301R^ mice.

Interestingly, these female-specific phenotypes were reverted by a single progestin treatment, implying that the female hormone cycle contributes to the altered behaviors noted in α_2_^+/G301R^ females compared to WT females and male α_2_^+/G301R^ mice. Intriguingly, this coincides with the influence of ovarian hormones on migraines, and the higher prevalence of migraines in women compared to men (Vos et al., 2012).

N-Methyl-D-aspartic acid or N-Methyl-D-aspartate (NMDA)-receptor antagonists also reverted specific α_2_^+/G301R^ phenotypes including hypoactivity, OCD-like behaviors and increased acoustic stimuli startle response. This indicates that the glutamatergic system is affected by the mutated α_2_ isoform. In support of this, α_2_^G301R/G301R^ mice had elevated glutamate levels in all major brain regions. Mixed astrocyte-neuron cultures from α_2_^G301R/G301R^ mice displayed a significant reduction in glutamate uptake compared to similar cultures from WT mice (Bøttger et al., 2016). This further links the female sex hormone cycle and the glutamate system to the comorbid psychiatric manifestations of FHM2.

## Cortical spreading depression

A key characteristic of FHM2 is the appearance of the aura phenomenon prior to the onset of the hemiplegic migraine. It is widely accepted that cortical spreading depression (CSD) is the molecular mechanism behind aura (Lauritzen et al., 2011). CSD is a short-lasting wave of neuronal and glial cell hyperactivity followed by a wave of long-lasting depression of neuronal activity (Pietrobon and Moskowitz, 2013). During the intense neuronal activity, excess K^+^ ions and glutamate are released into the extracellular space. The inefficient removal of K^+^ and glutamate by astrocytes can lead to wide-reaching depolarization that depresses further neuronal firing (Lauritzen et al., 2011; Pietrobon and Moskowitz, 2013). The CSD usually begins in the visual cortex and propagates slowly over the cortex at a rate of 2–5 mm/min (Pietrobon and Moskowitz, 2013).

*In vivo* CSD recordings revealed that α_2_^+/W887R^ mice were more susceptible to CSD. This is based on a 35% decrease in induction threshold relative to WT littermates (Leo et al., 2011). Furthermore, induced CSD events in the α_2_^+/W887R^ mice had a higher propagation velocity compared to WT littermates (Leo et al., 2011). The duration of triggered CSD events, however, was not altered between α_2_^+/W887R^ mice and littermates (Leo et al., 2011). Electrocorticography in α_2_^+/G301R^ mice, compared to WT littermates, revealed a prolonged recovery phase following CSD (Bøttger et al., 2016). Further CSD assays are necessary to conclude the effects of the G301R mutation on CSD.

It is difficult to compare the CSD studies of the two α_2_-KI mouse models, due to differing parameter and assay conditions used in each study. Potassium acetate, for example, was used for CSD induction in the right somatosensory cortex in α_2_^+/G301R^ mice, whereas incremental electrical current stimuli were delivered in the occipital cortex for the α_2_^+/W887R^ mice. Moreover, the different use of anesthetics (α-chloralose versus urethane) affects the data collection, as previous studies of other anesthetics were shown to influence the CSD induction threshold and to propagate speed differently (Kudo et al., 2013). Furthermore, only male mice were used in the α_2_^+/G301R^ study (Bøttger et al., 2016), whereas a mixed sex population was used for the α_2_^+/W887R^ study (Leo et al., 2011). In particular, considering the female specific phenotypes of the α_2_^+/G301R^, it appears that sex and specific sex hormones play an important role in FHM2. To sufficiently compare these two models and the CSD underlying FHM2, a comparable study using the same CSD induction method, same anesthetics and a focus on sex-specific phenotypes is required.

The CSD abnormalities observed in both FHM2 models are comparable with two FHM1 knock-in mouse models of which revealed increased susceptibility to CSD compared to WT mice. This further supports CSD as an important symptom of migraines and FHM in general (van den Maagdenberg et al., 2004, 2010). To further emphasize the importance of sex and CSD, it was found that female FHM1 KI mice were more susceptible to CSD than male KI mice (Eikermann-Haerter et al., 2009). This sex difference was abolished with ovariectomy and senescence, further solidifying the role of the female sex hormone cycle, besides genetics, in the susceptibility to CSD (Eikermann-Haerter et al., 2009).

## Perspectives on the use of FHM2 mouse models

Migraines affect nearly 15% of the world population, and while FHM2 is a relatively rare migraine form, further insight into FHM2 will lead to a broader understanding of migraines in general (Vos et al., 2012). Animal models of human genetic disorders are important tools for investigators in elucidating the molecular and physiological deficits leading to the disorder. As discussed in this review, much has already been learned from existing *Atp1a2* animal models (Ikeda et al., 2003, 2004; Lingrel et al., 2007; Moseley et al., 2007; Leo et al., 2011; Bøttger et al., 2016). The continued work with existing models and generating of new models with different FHM2 mutations will expand our understanding of FHM2. Further study of different FHM2 mouse models together with clinical descriptions of comparable FHM2 patients will shed light on any genotype-phenotype correlations, a critical aspect when studying a disorder with varying symptoms, severity, and different functional consequences of mutations.

FHM2 mouse models will be essential for drug development, drug screening and drug testing for treatment of FHM2 patients. A potential drug target for FHM2 is the Na^+^/K^+^-ATPase itself because lost pump function and/or decreased/altered pump activity is the main cause of FHM2 (Bøttger et al., 2012). Since all FHM2 patients are heterozygous, targeting the unmutated α_2_ isoform with activators could potentially overcome this haplodeficiency. In support of this therapeutic strategy, a recent report showed that crossing a *Atp1a3* mouse model (*Myskin*) for Alternating Hemiplegia of Childhood (AHC) with a mouse model overexpressing a WT *Atp1a3* allele could rescue the mutant phenotype through increased α_3_ pump activity (Kirshenbaum et al., 2016). Modulating Na^+^/K^+^-ATPase is also a desirable therapeutic strategy, due to this molecules' localization in the plasma membrane and well-regulated function. The mechanism of regulation is via factors (e.g., the cardiac glycoside ouabain) binding to the extracellular part of the pump.

An important aspect of *Atp1a2* deficits appears to be the resulting changes to the glutamatergic system. These changes are manifested through molecular and histological interactions between the mutant α2-subunit isoform and a glutamate transporter (i.e., EAAT1 or EAAT2) or through reduced glutamate uptake in cells with limited pump activity. Our data suggests that future studies of mouse models with elevated glutamate levels (e.g., α_2_-KO and -KI mice), which exhibit reversion of key specific α_2_-KI phenotypes when targeted with NMDA receptor antagonists (Cholet et al., 2002; Ikeda et al., 2003; Rose et al., 2009; Bøttger et al., 2016), might prove beneficial as a preclinical model to test drug targets within the glutamatergic system in FHM2 patients.

## Author contributions

All authors listed, have made substantial, direct and intellectual contribution to the work, and approved it for publication.

## Funding

TI and KL were supported by a grant from the Danish National Research Foundation (DNRF) (PUMPKIN DNRF85). TI was further supported by the Health Faculty, Aarhus University.

### Conflict of interest statement

The authors declare that the research was conducted in the absence of any commercial or financial relationships that could be construed as a potential conflict of interest.
